# Frailty and Dementia: Differences in Health Care Utilization and Costs

**DOI:** 10.1093/geroni/igab046.2996

**Published:** 2021-12-17

**Authors:** Stephanie Denise Sison, Gahee Oh, Sandra Shi, Brianne Olivieri-Mui, Ellen McCarthy, Dae Kim

**Affiliations:** 1 Beth Israel Deaconess Medical Center / VA Boston New England GRECC, Boston, Massachusetts, United States; 2 Hinda and Arthur Marcus Institute for Aging Research, Roslindale, Massachusetts, United States; 3 Hebrew SeniorLife, Harvard Medical School, Roslindale, Massachusetts, United States; 4 Hebrew SeniorLife, roslindale, Massachusetts, United States; 5 Hebrew SeniorLife, Boston, Massachusetts, United States

## Abstract

Frailty and dementia are associated with poor health outcomes and increased health care utilization. A more nuanced understanding of this dynamic may be useful in improving care and developing policies. This retrospective cohort study was conducted using 5% random sample of Medicare fee-for-service beneficiaries (n=1,132,367; mean age 76.2 years; 57.9% female) in 2014-2016. We compared average 1-year home time (number of days alive outside of the hospital and SNF), mean total cost per beneficiary, and number of incident ICU stays per 100 person-years (PY) across four groups: frailty and dementia, dementia alone, frailty alone or neither. Frailty and dementia were identified using validated claims-based algorithms. We also determined differences in costs per group across different regions within the United States. Beneficiaries with both frailty and dementia had a high 1-year mortality rate of 21.9% (vs. dementia alone [9.7%], frailty alone [9.4%] or neither [2.1%]), while having less home time (306 days; difference of 36 days, 31 days, and 53 days, respectively), and more incident ICU stays per 100 PY (29.9 vs 9.5, 25.8, and 5.6, respectively). Mean total costs for beneficiaries with both was $26,030 compared to other groups ($12,096, $24,693, and $9,029, respectively). Across the United States, range of costs varied the most for beneficiaries with both frailty and dementia ($13,244-31,987 vs $4,621-15,364, $20,090-30,965, and $7,672-10,450, respectively). Increase in health care utilization and wide geographic variation in costs associated with patients with frailty and dementia suggests room for improvement in health care delivery to improve outcomes of this group.

